# 14-3-3ζ mediates an alternative, non-thermogenic mechanism in male mice to reduce heat loss and improve cold tolerance

**DOI:** 10.1016/j.molmet.2020.101052

**Published:** 2020-07-12

**Authors:** Kadidia Diallo, Sylvie Dussault, Christophe Noll, Angel F. Lopez, Alain Rivard, André C. Carpentier, Gareth E. Lim

**Affiliations:** 1 Département de médecine, Université de Montréal, Montréal, QC, Canada; 2 Axe cardio-métabolique, Centre de Recherche du Centre Hospitalier de l’Université de Montréal (CRCHUM), Montréal, Québec, Canada; 3 Département de médecine, Université de Sherbrooke, Sherbrooke, QC, Canada; 4 Centre de Recherche du Centre Hospitalier de l’Université de Sherbooke (CHUS), Sherbrooke, QC, Canada; 5 Centre for Cancer Biology, SA Pathology and University of South Australia, Adelaide, South Australia, Australia

**Keywords:** 14-3-3 proteins, 14-3-3ζ, Beiging, Vasoconstriction, Adaptive thermogenesis

## Abstract

**Objective:**

Adaptive thermogenesis, which is partly mediated by sympathetic input on brown adipose tissue (BAT), is a mechanism of heat production that confers protection against prolonged cold exposure. Various endogenous stimuli, for example, norepinephrine and FGF-21, can also promote the conversion of inguinal white adipocytes to beige adipocytes, which may represent a secondary cell type that contributes to adaptive thermogenesis. We previously identified an essential role of the molecular scaffold 14-3-3ζ in adipogenesis, but one of the earliest, identified functions of 14-3-3ζ is its regulatory effects on the activity of tyrosine hydroxylase, the rate-limiting enzyme in the synthesis of norepinephrine. Herein, we examined whether 14-3-3ζ could influence adaptive thermogenesis via actions on BAT activation or the beiging of white adipocytes.

**Methods:**

Transgenic mice over-expressing a TAP-tagged human 14-3-3ζ molecule or heterozygous mice without one allele of *Ywhaz*, the gene encoding 14-3-3ζ, were used to explore the contribution of 14-3-3ζ to acute (3 h) and prolonged (3 days) cold (4 °C) exposure. Metabolic caging experiments, PET-CT imaging, and laser Doppler imaging were used to determine the effect of 14-3-3ζ over-expression on thermogenic and vasoconstrictive mechanisms in response to cold.

**Results:**

Transgenic over-expression of 14-3-3ζ (TAP) in male mice significantly improved tolerance to acute and prolonged cold. In response to cold, body temperatures in TAP mice did not decrease to the same extent when compared to wildtype (WT) mice, and this was associated with increased UCP1 expression in beige inguinal white tissue (iWAT) and BAT. Of note was the paradoxical finding that cold-induced changes in body temperatures of TAP mice were associated with significantly decreased energy expenditure. The marked improvements in tolerance to prolonged cold were not due to changes in sensitivity to β-adrenergic stimulation or BAT or iWAT oxidative metabolism; instead, over-expression of 14-3-3ζ significantly decreased thermal conductance and heat loss in mice via increased peripheral vasoconstriction.

**Conclusions:**

Despite being associated with elevations in cold-induced UCP1 expression in brown or beige adipocytes, these findings suggest that 14-3-3ζ regulates an alternative, non-thermogenic mechanism via vasoconstriction to minimize heat loss during cold exposure.

## Highlights

• 14-3-3ζ over-expression in male mice improves tolerance to acute and prolonged cold.
• Increasing 14-3-3ζ expression promotes beiging of inguinal white adipose tissue.
• Cold-induced changes in body temperature can be dissociated from energy expenditure.
• 14-3-3ζ-dependent decreases in heat loss are associated with vasoconstriction.

## Graphical abstract

**Figure:**
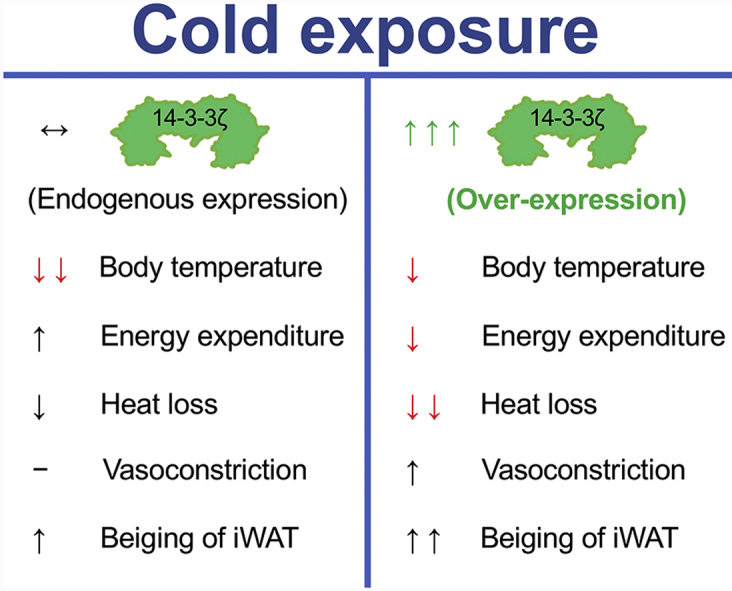

## 1 Introduction

Homeothermy is the maintenance of a stable, internal body temperature following changes in environmental temperature [1], and in the context of hypothermia, mammals have evolved various thermogenic mechanisms, ranging from skeletal muscle shivering to non-shivering, adaptive thermogenesis, to protect against cold [[1], [2], [3], [4], [5]]. Shivering, which is short in duration, is the fast contraction of skeletal muscles during which heat is released via the hydrolysis of ATP [6]. Another rapid mechanism to regulate body temperature is vasoconstriction, which helps to mitigate heat loss [7], but its relative contribution to homeothermy in rodents is often underappreciated.

The sympathetic nervous system (SNS) is the primary regulator of adaptive thermogenesis because the release of norepinephrine from efferent nerves activates β-adrenergic receptors on the surface of brown adipocytes in rodents to trigger heat production [8]. Long-term cold exposure can also induce the recruitment of brown-like adipocytes in inguinal white adipose tissue (iWAT) through beiging [9,10]. Brown and beige adipocytes are distinct thermogenic fat cells rich in uncoupling protein-1 (UCP1), which uncouples the proton gradient from ATP synthesis to produce heat during fatty acid oxidation [8,9,11]. Although the contributions of BAT to thermogenesis have been well-established, the relative contributions of beige adipocytes to thermogenesis are unclear [12]. However, in the context of adrenergic stress, such as severe burns, detectable increases in beige adipocyte thermogenic activity have been reported [13,14]. Recently, emphasis on investigating the therapeutic potential of treating diabetes and obesity by activating beige and brown adipocytes has been proposed [9,15], but the mechanisms underlying brown and beige adipocytes' development and function remain partially understood.

14-3-3ζ is a member of the 14-3-3 scaffold protein family, which are highly conserved serine and threonine binding proteins present in all eukaryotes [[16], [17], [18], [19]]. They bind to a diverse number of enzymes, signaling proteins, and transcription factors and have been implicated in the regulation of numerous cellular processes, including proliferation, protein trafficking, and apoptosis [17,18]. Recently, we have reported various contributions of 14-3-3ζ to whole body metabolism because it was found to be a critical regulator of glucose metabolism and adipogenesis [[20], [21], [22]]. Interactions of 14-3-3 proteins with enzymes have been documented to exert positive or inhibitory effects on their activities. For example, the binding of 14-3-3 proteins to RAF-1 or PKA potentiates their kinase activity [23,24]; by contrast, interactions of DYRK1A with 14-3-3 proteins can attenuate kinase function [25]. Notably, one of the first ascribed functions of 14-3-3 proteins is their regulation of tyrosine (TH) and tryptophan (TPH) hydroxylases, both of which are rate-limiting enzymes involved in the synthesis of norepinephrine and serotonin, respectively [26]. Furthermore, norepinephrine and serotonin have been demonstrated to respectively stimulate and inhibit adaptive thermogenesis and beiging [1,[27], [28], [29]]. Therefore, because of the ability of 14-3-3ζ to regulate the activities of tyrosine hydroxylase TH and TPH, we hypothesized that 14-3-3ζ may have critical roles in the development and function of beige and brown adipocytes, influencing adaptive thermogenesis.

In this study, we report on the outcome of reducing or increasing 14-3-3ζ expression in the context of tolerance to acute and prolonged cold stress. Transgenic mice over-expressing 14-3-3ζ were protected from cold exposure, and in the context of prolonged cold stress, 14-3-3ζ over-expression was associated with the ability to better defend against cold-induced decreases in body temperature. This was accompanied by paradoxical decreases in energy expenditure, despite a lack of differences in oxidative metabolism in BAT and various tissues when compared to wildtype mice. No differences in sensitivity to β-adrenergic stimuli or sympathetic activity were detected when 14-3-3ζ levels were increased. Strikingly, mice over-expressing 14-3-3ζ displayed decreased thermal conductance, or heat loss, due to elevated vasoconstriction. Collectively, our data demonstrate that 14-3-3ζ over-expression improves cold tolerance by activating a non-thermogenic mechanism to mitigate heat loss in the context of cold tolerance. Furthermore, results from this study highlight the need to consider the contributions of non-thermogenic mechanisms to cold tolerance or adaptation and caution against invoking solely increased thermogenesis to explain tolerance to prolonged cold exposure.

## 2 Methods

### 2.1 Animal studies

Wildtype (WT) and 14-3-3ζ heterozygous (HET) mice were on a C57Bl/6J background, and transgenic (TAP) mice over-expressing a TAP-tagged human 14-3-3ζ molecule were on a CD-1 background [22,30]. Mice were housed in a temperature-controlled (22 °C) animal facility on a 12-hour light/dark cycle in the Centre de Recherche du Centre Hospitalier de l’Université de Montréal (CRCHUM). Mice had *ad libitum* access to water and standard chow (TD2918, Envigo, Huntingdon, United Kingdom), and all animal studies were performed in accordance with the Comité Institutionnel de Protection des Animaux of the CRCHUM.

For acute cold challenges, 12-week-old mice were individually caged and fasted for 4 h before and during a 3-hour challenge at 4 °C with *ad libitum* access to water. Body temperature was measured with a physio-suit rectal probe (Kent Scientific, Torrington, CT, USA). For prolonged cold challenges, mice were housed in Comprehensive Lab Animal Monitoring System (CLAMS, Columbus Instruments Columbus, OH, USA) metabolic cages or a cold room for 3 days at 4 °C. The β3 adrenergic agonist CL316,243 (Sigma Aldrich, St Louis, MO, USA) was diluted in saline 0.9%, and mice received daily intraperitoneal injections of either saline 0.9% or CL316,243 (1 mg/kg) for 7 days. Body composition (lean and fat mass) was determined by using an echo MRI (EchoMRI™, Houston, TX, USA).

### 2.2 Cell culture

The immortalized UCP1-luciferase (UCP1-Luc) adipocyte cell line was provided by Dr. Shingo Kajimura (Diabetes Center, University of California- San Francisco) [31]. Cells were grown in 25 mM glucose DMEM media (Thermo Fisher Scientific, Waltham, MA, USA), supplemented with 10% fetal bovine serum (FBS; Thermo Fisher Scientific) and 1% streptomycin (Thermo Fisher Scientific) and grown at 37 °C, 5% CO_2_. UCP1-Luc cells were induced to differentiation into brown adipocytes with a cocktail containing 5 μg/ml insulin (Sigma Aldrich), 0.5 mM 3-Isobutyl-1-methylxanthine IBMX (Sigma Aldrich), 1 μM dexamethasone (Sigma Aldrich), 0.125 mM indomethacin (Sigma Aldrich), and 1 nM 3,3′,5-Triiodo-l-thyronine T3 (Sigma Aldrich) for 2 days, followed by a maintenance medium containing 5 μg/ml insulin and 1 nM T3 on day 3, and DMEM/FBS growth medium on day 5. Lipofectamine RNAiMax (Invitrogen, Carlsbad, CA, USA) and Silencer Select siRNA against *Ywhaz* (gene encoding 14-3-3ζ) and a scrambled, control siRNA (Ambion, Austin, TX, USA) were used to knockdown 14-3-3ζ protein expression, as previously described [22].

### 2.3 Immunoblotting

Inguinal (iWAT) and gonadal (gWAT) white adipose tissues and interscapular brown adipose tissue (BAT) were homogenized in RIPA lysis buffer (50 mM β glycerol phosphate, 10 mM Hepes, pH = 7.4, 70 mM NaCl, 1% Triton X-100, 2 mM EGTA, 1 mM Na_3_VO_4_, 1 mM NaF), supplemented with protease and phosphatase inhibitors. Lysates were centrifuged at 13,000 rpm for 15 min at 4 °C, the supernatant was collected, and protein concentration was determined by using Bio-Rad protein assay dye reagent (Bio-Rad, Hercules, CA, USA). Protein samples were resolved by SDS-PAGE, transferred to PVDF membranes, and blocked with I-block (Applied Biosystems, Foster City, CA, USA) for 1 h at room temperature, followed by overnight incubation at 4 °C with primary antibodies against UCP1 (1:1000, R&D systems, Minneapolis, MN, USA), 14-3-3ζ (1:1000 Cell Signaling, Danvers, MA, USA), β-Actin (1:10000, Cell Signaling), β-Tubulin (1:1000, Cell Signaling), and TH (1:1000, Millipore, Bilerica, MA, USA). The next day, membranes were washed and incubated with horseradish peroxidase-conjugated secondary antibodies (1:5000, Cell Signaling) for 1 h at room temperature. Immunoreactivity was detected by chemiluminescence with a ChemiDoc system (Bio-Rad). Information for each antibody is in Supplemental Table 1.

### 2.4 Histology and immunofluorescence

IWAT, gWAT, and BAT were excised and fixed in 4% PFA (Sigma Aldrich) for 7 days and stored in 70% ethanol before embedding in paraffin. Sections of 5 μm thickness were deparaffinized, rehydrated, and stained with hematoxylin (Sigma Aldrich) and eosin (Sigma Aldrich). Alternatively, slides were stained with a UCP1 antibody (1:250, Abcam, Cambridge, United Kingdom), followed by an HRP-conjugated secondary antibody for DAB labeling (Cell signaling). Images were taken at 20X (Nikon Eclipse Ti2, Nikon Instruments Inc, Melville, NY, USA).

For immunofluorescence, sections were stained for perilipin (1:400, Cell Signaling). Antigen retrieval was performed with 10 mM sodium citrate buffer (Sigma Aldrich) at pH = 6–6.2 for 15 min at 95 °C. Sections were blocked for 1 h at room temperature with PBS-T (0.1% Triton, 5% normal donkey serum) and incubated overnight in PBS-T at 4 °C with primary antibodies. Alexa Fluor 594-conjugated secondary antibodies (Jackson Immuno-research laboratories, Inc, West Grove, PA, USA) were incubated for 1 h at room temperature, and slides were mounted in Vectashield containing DAPI (Vector Laboratories, Burlingame, CA, USA). Total adipocyte number and area were counted from 8 to 10 images per mouse and then measured by using Cell Profiler software (CellProfiler Analyst, Stable (2.2.1) [32]. All immunofluorescence images were acquired with an EVOS FL microscope (Thermo Fisher Scientific). Information for each antibody is in Supplemental Table 1.

### 2.5 RNA isolation and quantitative PCR

Total RNA was isolated from cells and tissues by using the RNeasy Mini kit or the RNeasy Plus Mini Kit (Qiagen, Montréal, Québec, Canada), respectively, and stored at −80 °C. Reverse transcription was performed with the High Capacity cDNA Reverse Transcription Kit (Applied Biosystems) or the Superscript VILO Kit (Invitrogen), in accordance with the manufacturer's instructions. Gene expression was analyzed by quantitative PCR (qPCR) with SYBR Green chemistry (PowerUp SYBR, ThermoFisher Scientific) on a QuantStudio 6 Real-Time PCR machine (Applied Biosystems, Life Technologies, Carlsbad, CA, USA). All quantitative PCR results were expressed as relative gene expression (rel. exp.), which is the gene of interest normalized to the housekeeping gene, *Hprt,* by using the 2^−ΔC(t)^ method [19,22,33]. For a complete list of primers and their respective sequences, please see Supplemental Table 2.

### 2.6 Metabolic phenotyping

Male WT and TAP mice at 16 weeks of age received abdominal surgery to implant a temperature probe 10 days before their placement in CLAMS. Bodyweight and body composition were measured before and after prolonged cold exposure by using an echo MRI on living, non-anesthetized mice. Mice were singly housed in CLAMS cages with *ad libitum* access to water and a normal chow diet and were maintained on a 12-hour light/dark cycle on the following schedule: 24 h at 22 °C for acclimatization, 24 h at 22 °C for basal measurements, and 72 h at 4 °C for the prolonged cold challenge. Food intake, respiratory exchange ratio, locomotor activity (beam breaks), energy expenditure (heat), and core body temperature (°C) were measured in real-time every 15–20 min. Following prolonged cold exposure, blood and tissues were collected and snap frozen for subsequent use. Thermal conductance, or the ease by which heat escapes into the environment, was calculated [[34], [35], [36]]: It was derived from the formula, C = EE/(Tb − Ta), where C = conductance, EE = energy expenditure, Tb = core temperature, and Ta = ambient temperature [34,35].

Cohorts of mice were administered intraperitoneal glucose tolerance tests (IPGTTs) after 6 h of fasting. IPGTTs were performed at 22 °C and after 3 days of 4 °C exposure. Mice were injected with glucose (2 mg/kg), and blood glucose was measured with a Contour Next glucose meter (Ascensia Diabetes Care, Basel, Switzerland). Circulating free fatty acids were measured from plasma samples by using the Wako NEFA-HR (2) assay kit (Wako Pure Chemical Industries LTD, Osaka, Japan), and circulating glycerol was measured from plasma samples by using the triglyceride and free glycerol reagents (Sigma Aldrich) as per the manufacturers' instructions. Circulating leptin (ALPCO, Salem, NH, USA) was measured from plasma samples, in accordance with the manufacturers' protocols. Norepinephrine (Rocky Mountain Diagnostics, Colorado Springs, CO, USA) was measured from iWAT and BAT tissue extracts following the manufacturers' instructions.

### 2.7 Laser Doppler imaging

Laser Doppler imaging was used to measure peripheral tail blood flow, as a surrogate for vasoconstriction [37]. In summary, female WT and TAP mice were anesthetized and tail perfusion was measured with the Laser Doppler Perfusion Imager System (Moor Instruments Ltd., Axminister, UK) [38,39]. Consecutive measurements were obtained from anesthetized mice by scanning the base of the tail. Color images were acquired, and flux values were measured by calculating the average perfusion signal. All experiments were performed at 22 °C, and anesthetized mice were placed on heating pads until the laser Doppler measurements were performed. Mice were sacrificed at the end of the study by exsanguination under anesthesia (isoflurane).

### 2.8 Positron emission tomography/computed tomography (PET/CT) imaging

μPET/CT experiments were approved by the Ethical Committee for Animal Care and Experimentation of the Université de Sherbrooke. Mice underwent a randomized, crossover thermoneutrality (30 °C) *vs.* cold exposure (4 °C) for 3 days, with a 1-week washout period in between, before sequential μPET dynamic imaging with [^11^C]-acetate and [^18^F]-FDG (2-[^18^F]fluoro-2-deoxy-glucose) after 6 h of fasting. μPET/CT experiments were performed under anesthesia (isoflurane 2.0%, 1.5 L.min^−1^), delivered to the animal through a nose cone. After cold exposure, mice were injected with the β3-agonist CL316,243 (2 mg/kg) before the PET tracer injection to maintain cold-induced BAT stimulation, as previously published [40]. All PET tracers were injected through the tail vein, and imaging was performed with the avalanche photodiode-based small animal μPET scanner (LabPET/Triumph, Gamma Medica, Northridge, CA) in the Sherbrooke Molecular Imaging Centre (*Centre de recherche du Centre Hospitalier Universitaire de Sherbrooke,* Sherbrooke, QC, Canada). Anesthetized mice were placed on the scanner bed and positioned with the heart centered within the field-of-view of the scanner. A bolus of [^11^C]-acetate (10 MBq, in 0.2 mL of 0.9% NaCl) was injected intravenously followed by a 15-min whole body dynamic μPET acquisition. Next, a bolus of [^18^F]-FDG (10 MBq, in 0.1 mL of 0.9% NaCl) was injected and a 20-min whole body dynamic PET acquisition was performed. Residual [^11^C]-acetate activity during [^18^F]-FDG acquisition was corrected by acquiring a 60 s frame before the injection of [^18^F]-FDG, accounting for the disintegration rate of [^11^C]. Low dose CT scan imaging was performed by using the integrated X–O small animal CT scanner on the Triumph platform, comprising a 40 W X-ray tube with a 75 μm focal spot diameter and a 2240 × 2368 CsI flat panel X-ray detector. All images were analyzed as described previously [40]. The tissue (except BAT) blood flow index [K_1_ in min^−1^ of [^11^C]-acetate and oxidative metabolism index (K_2_ in min^−1^ of [^11^C]-acetate) were estimated from [^11^C]-acetate by using a three-compartment model [41]. BAT K_1_ and K_2_ were estimated from [^11^C]-acetate by using a new multicompartment model specific to BAT [42]. Tissue glucose fractional extraction (Ki – *i.e.*, the fraction of circulating glucose taken up by tissues over time) was determined by using the Patlak graphical analysis of [^18^F] activity. Tissue glucose uptake (Km) was determined by multiplying Ki by the plasma glucose concentration.

### 2.9 Statistical analysis

Data are presented as mean ± standard error. Statistical analyses were performed with GraphPad Prism 8 (GraphPad Software) by using Student's t test or one- or two-way ANOVA, followed by appropriate *post hoc* tests. Statistical significance was achieved when p < 0.05.

## 3 Results

### 3.1 Partial deletion of 14-3-3ζ does not affect acute cold tolerance

To understand whether 14-3-3ζ could influence cold tolerance, we started by examining the outcome of partial deficiency of *Ywhaz,* the gene encoding 14-3-3ζ, in the context of acute exposure to cold. We have reported that homozygous 14-3-3ζ knockout mice weighed significantly less than WT mice due to reduced fat mass, which could affect their ability to tolerate cold [22,43,44]; thus, mice heterozygous (HET) for *Ywhaz* were used. In male WT and HET mice challenged with acute cold (4 °C) for 3 h, no differences in body weights were detected between WT and HET mice before and after the acute cold challenge (Figure S1A,B). Furthermore, both groups displayed similar decreases in rectal temperature throughout the entire challenge (Figure S1C,D). Similar results were observed in female mice (Figures S2A–C). Surprisingly, quantitative PCR analyses of *Ywhaz,* the gene encoding 14-3-3ζ, showed no differences in mRNA transcript levels in HET mice, suggesting the presence of endogenous, compensatory mechanisms to maintain levels of *Ywhaz* mRNA levels comparable to WT mice (Figure S1E,F and Figure S2D,E) [45,46], and this could account for the lack of differences in acute cold tolerance. Nonetheless, acute cold exposure significantly increased *Ywhaz* and *Ucp1* mRNA levels in BAT of WT and HET mice (Figure S1E, Figure S2D). In iWAT, levels of *Ywhaz* mRNA were significantly increased by cold exposure in WT and TAP mice, but no differences were observed in the expression of *Ucp1* and the beige-selective gene *Tmem26* (Figure S1F, Figure S2E), indicating that 3 h of cold exposure was insufficient to induce beiging [9,10].

To further determine if 14-3-3ζ is necessary for *Ucp1* mRNA expression in brown adipocytes, we used the UCP1-luciferase adipocyte cell line, an *in vitro* model of brown adipocytes [31]. Transient knockdown of *Ywhaz* by siRNA did not affect isoproterenol-induced *Ucp1* mRNA expression, which suggests that 14-3-3ζ is not required for *Ucp1* expression (Figure S1G,H). Taken together, these *in vitro* and *in vivo* data demonstrate that reducing 14-3-3ζ expression does not affect cold tolerance or *Ucp1* gene expression.

### 3.2 14-3-3ζ over-expression provides tolerance to acute cold

**Figure 1:**
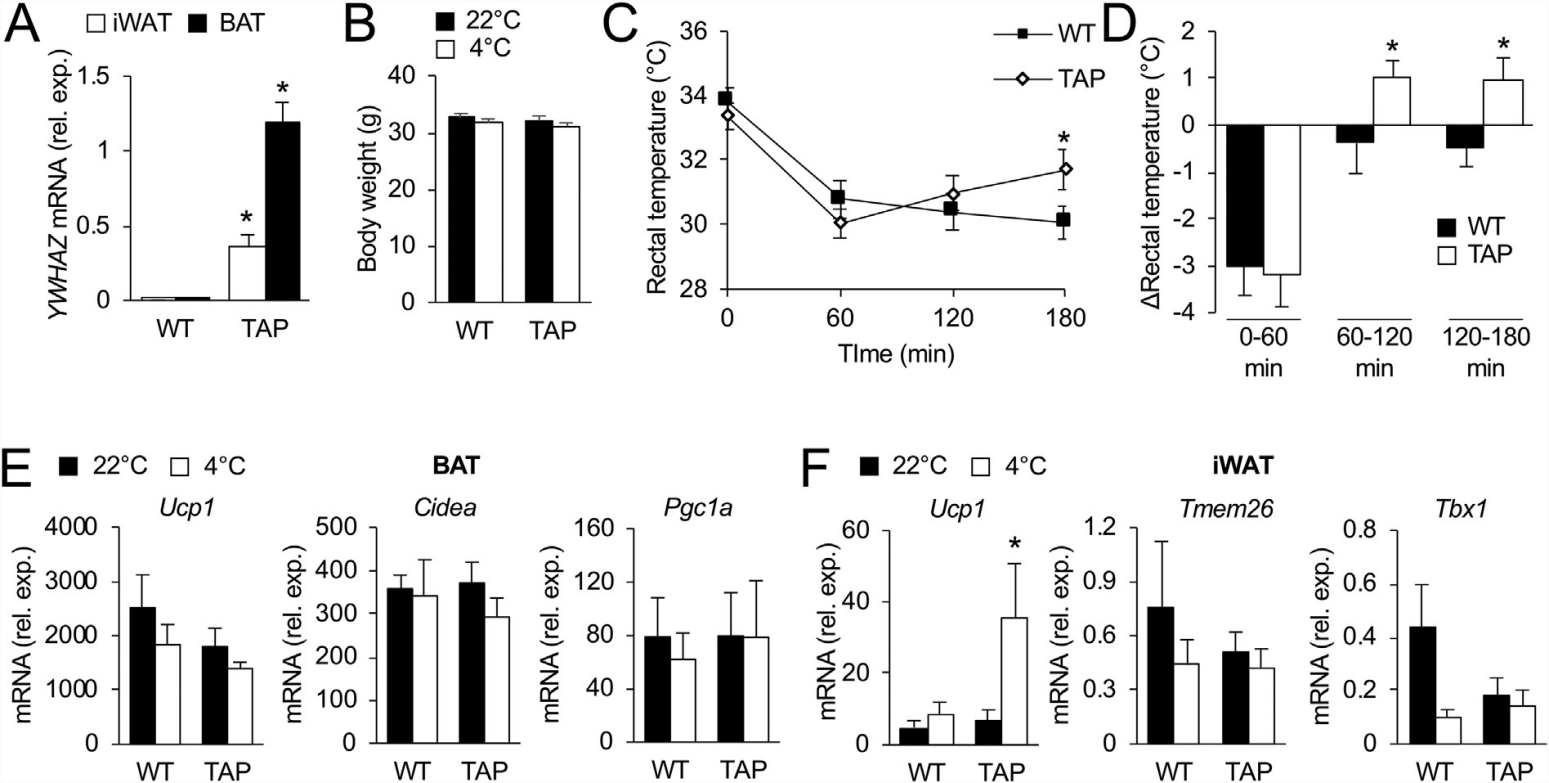
**Over-expression of 14-3-3ζ enhances tolerance to acute cold in male mice. (A)** Quantitative PCR was used to measure *YWHAZ* (gene encoding human 14-3-3ζ) in the iWAT and BAT of TAP mice (n = 3 per group, ∗: p < 0.05). **(B**–**D)** Body weights (B), rectal temperatures (C), and change (Δ) in rectal temperature (D) WT and TAP mice were obtained before, during, and at the end of the 3-hour cold challenge (n = 7 mice per group; ∗: p < 0.05 when compared to WT mice at same time point). **(E,F)** Expression of brown-selective (E) and beige-selective (F) genes from BAT and iWAT, respectively, at room temperature (22 °C) and after 3 h of cold exposure (n = 7 per group, ∗: p < 0.05 when compared to 22 °C). Data are represented as mean ± SEM.

We next examined if increasing 14-3-3ζ expression could affect cold tolerance. Twelve-week-old transgenic (TAP) mice over-expressing a TAP-tagged human 14-3-3ζ molecule were challenged with acute cold (4 °C) exposure for 3 h (Figure 1A, Figure S1A). In male mice, no differences in body weight were detected before or after cold exposure (Figure 1B); however, male TAP mice displayed a significant restoration of rectal temperature, signifying improved tolerance to acute cold exposure (Figure 1C,D). We observed no differences in body weights in female TAP mice before or after cold exposure (Figure S3A) and no differences in rectal temperatures throughout the acute cold challenge (Figure S3B,C). In BAT of male mice, acute cold exposure had no effect on the expression of *Ucp1*, *Pgc1a,* or *Cidea,* but BAT from female mice exhibited increases in *Pgc1a* mRNA after cold exposure (Figure 1E, Figure S3D). By contrast, significant increases in *Ucp1* mRNA levels in iWAT of both male and female TAP mice after acute cold were detected (Figure 1F, S3E). Taken together, these data demonstrate that 14-3-3ζ over-expression in male mice is sufficient to improve tolerance to acute cold exposure.

### 3.3 Over-expression of 14-3-3ζ improves tolerance to prolonged cold exposure

**Figure 2:**
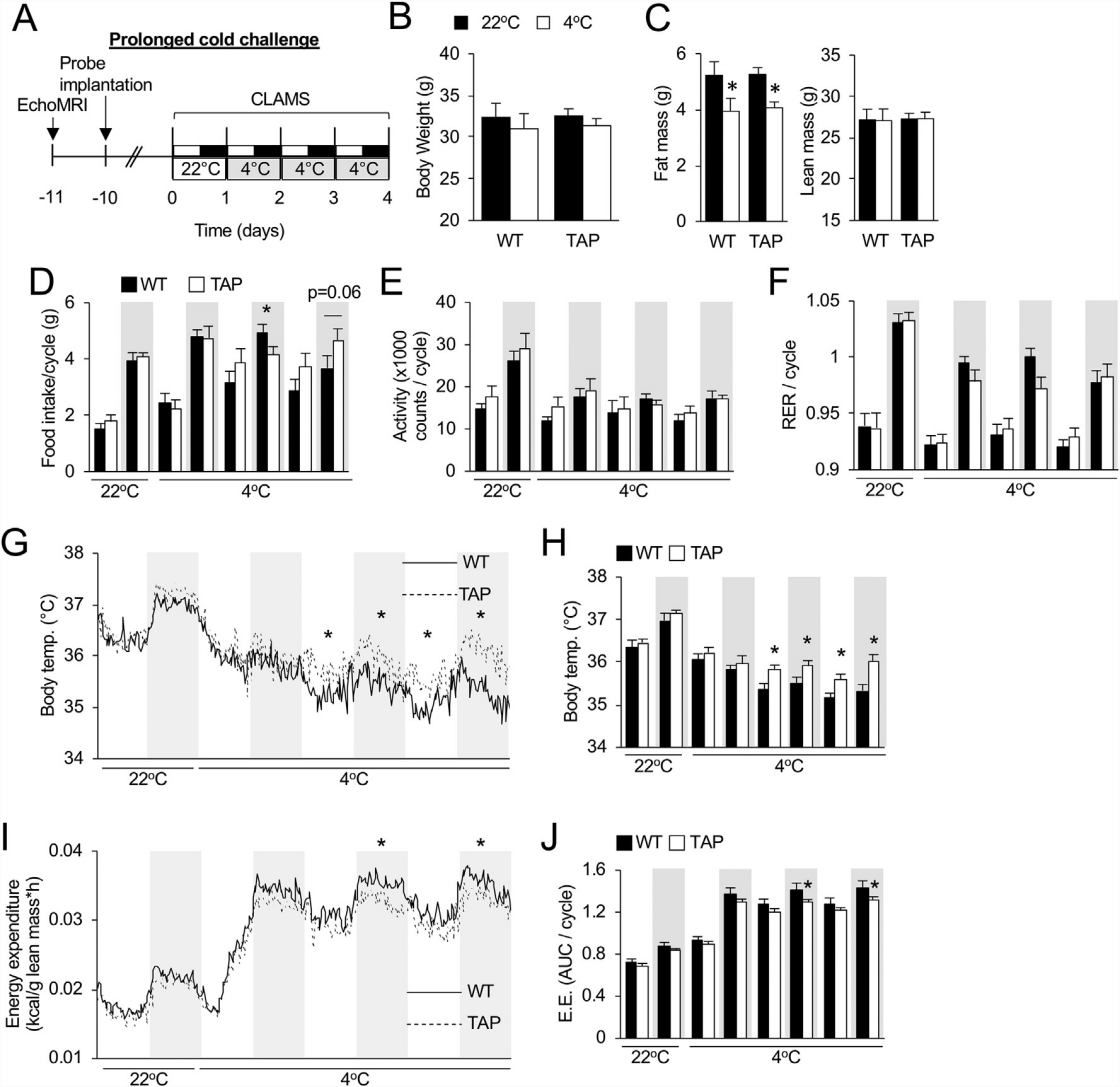
**Over-expression of 14-3-3ζ improves tolerance to prolonged cold exposure in male mice. (A)** Wildtype (WT) and 14-3-3ζ over-expressing (TAP) mice were implanted with a temperature probe 1 week before placement in CLAMS metabolic cages for 3 days at 4 °C. **(B,C)** Body weights (B) and lean and fat mass measurements by echo MRI (C) were obtained before and after the prolonged cold challenge. **(D**–**F)** During the light and dark cycles at 22 °C and 4 °C, food intake (G), locomotor activity (H), and RER were measured in WT and TAP mice. **(G**–**J)** Body temperature (G,H) and energy expenditure, relative to the lean mass of mice (I,J), were measured and are reported either as the average trace for all mice over the cold challenge (G, I) or as an average per light:dark cycle (H,J; n = 8 WT and n = 10 TAP mice; ∗: p < 0.05 when compared to WT). Data are represented as mean ± SEM.

Due to the improved tolerance to acute cold exposure in male TAP mice and the ability of cold to augment *Ucp1* mRNA levels in iWAT (Figure 1C,D, and F), male WT and TAP mice were subjected to a prolonged cold (4 °C) challenge of 3 days (Figure 2A). When compared to WT mice, TAP mice had similar body weights before cold exposure and lost comparable body weight after the challenge (Figure 2B). Additionally, fat and lean mass in WT and TAP mice before and after cold exposure were not different (Figure 2C). Food intake was increased in WT and TAP mice when the temperature was lowered from 22 °C to 4 °C, which is consistent with the need to supply necessary fuel as compensation for energy dissipated during thermogenesis [47], but no differences were observed between the groups (Figure 2D). Moreover, locomotor activity or RER was unchanged in the groups (Figure 2E,F). TAP mice were better able to defend against cold-induced decreases in core body temperatures because significant differences were detected between WT and TAP mice during the last 48 h of cold exposure (Figure 2G,H). Unexpectedly, significantly decreased energy expenditure during the last 2 dark phases of the cold challenge were observed (Figure 2I,J; Figure S4).

**Figure 3:**
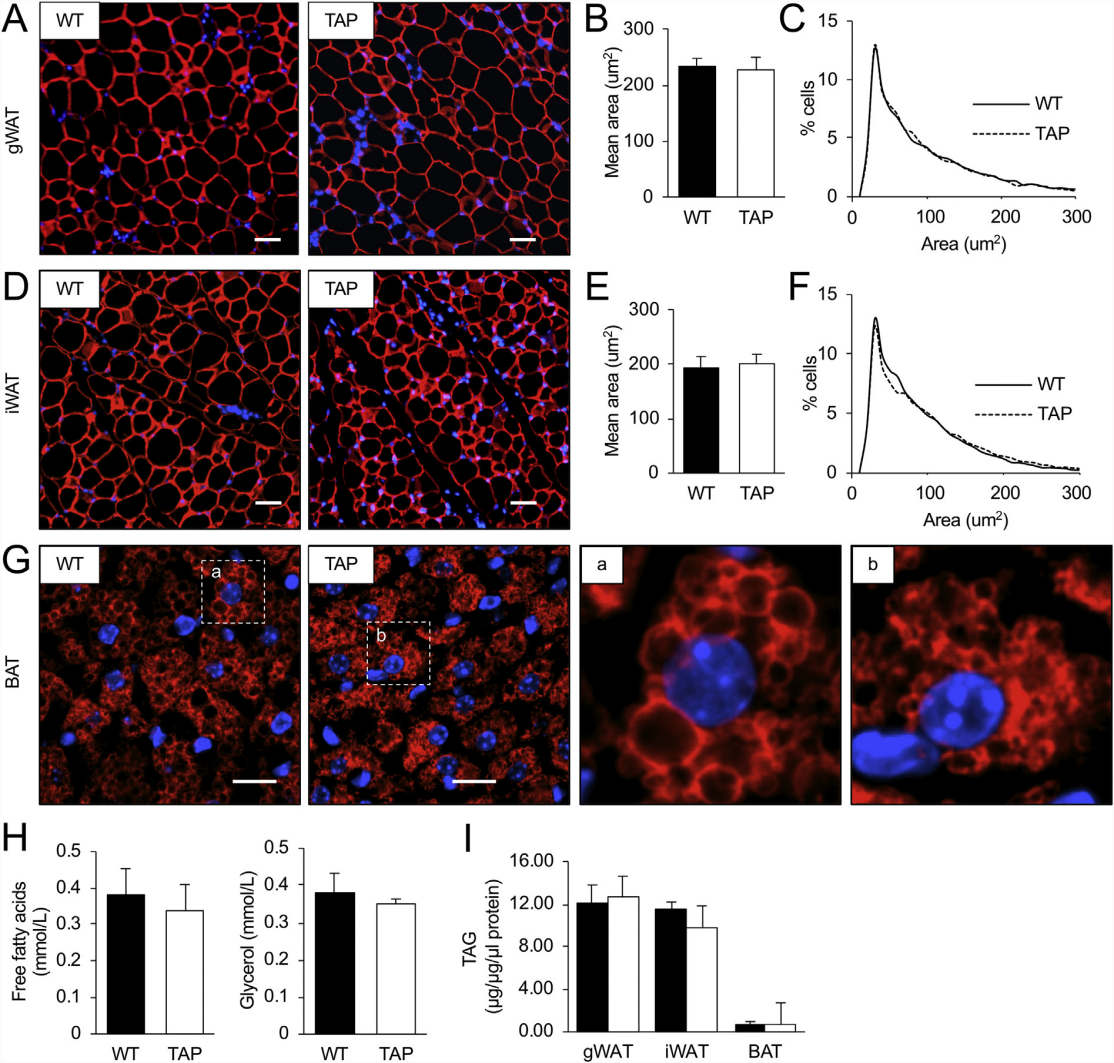
**Adipocyte size in gonadal or inguinal white adipose tissue is not affected by 14-3-3ζ over-expression following cold exposure. (A**–**F)** Immunofluorescent staining was used to examine adipocyte morphology (A,D), average size (B,E), and size distribution (C,F) in gonadal white adipose tissue (gWAT, A-C) and inguinal WAT (E,F) (representative images of n = 4–5 mice per group; scale bar = 200 μm). (**G)** Morphology of BAT was measured by immunostaining for perilipin. Inset images of brown adipocytes from WT (a) and TAP (b) male mice (representative images of n = 4–5 mice per group; scale bars = 100 μm). **(H,I)** Circulating free fatty acids and triglycerides (H) and total triacylglycerols in gWAT, iWATm, and BAT (I) of male WT and TAP mice were measured after 3 days of cold exposure (n = 4–5 per group). Data are represented as mean ± SEM.

Analysis of adipocyte morphometry revealed no differences in adipocyte size in iWAT or gonadal WAT (gWAT) of WT and TAP mice (Figure 3A–F). However, after prolonged cold exposure, distinct morphological differences were observed in BAT, whereby smaller lipid droplets were visible in TAP mice (Figure 3G). Despite these differences in the size of lipid droplets in BAT of TAP mice, circulating free fatty acids and glycerol levels after prolonged cold exposure were similar between WT and TAP mice (Figure 3H), and no differences in total triacylglycerol content in BAT were detected (Figure 3I).

### 3.4 Over-expression of 14-3-3ζ potentiates beiging of inguinal white adipocytes in response to prolonged cold exposure

**Figure 4:**
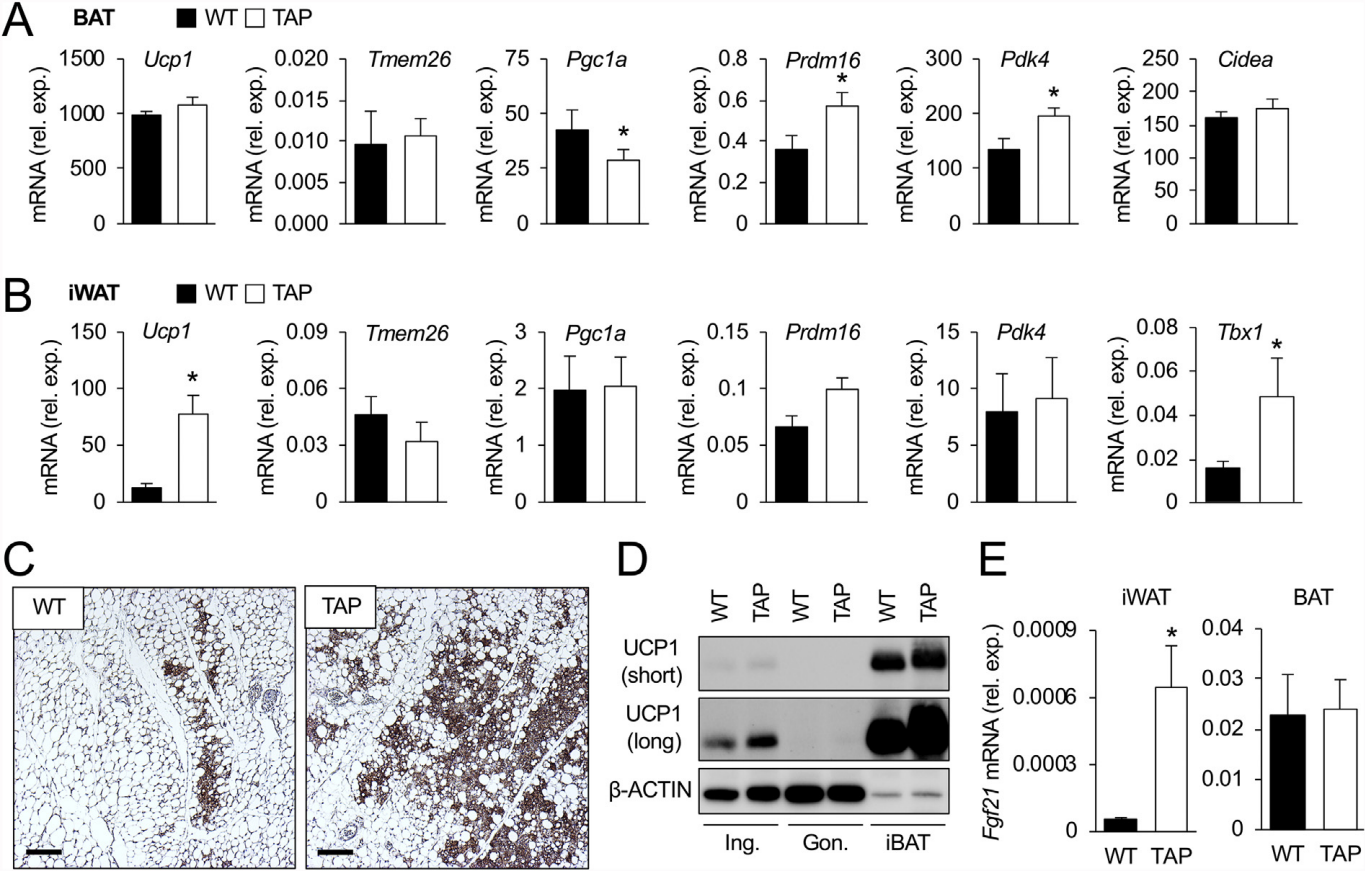
**Transgenic over-expression of 14-3-3ζ increases beiging of ingulnal white adipocytes in male mice. (A,B)** After 3 days of cold exposure, BAT (A) and inguinal white adipose tissue (iWAT, B) from wildtype (WT) and transgenic mice over-expressing 14-3-3ζ (TAP) were harvested, followed by quantitative PCR to measure genes associated with thermogenesis and beiging (n = 8 WT and 10 TAP; ∗: p < 0.05). **(C)** Immunohistochemistry was used to detect UCP1 immunoreactivity in paraffin embedded iWAT sections from WT or TAP mice exposed to prolonged cold (representative images for n = 8 WT and 10 TAP mice; scale bar = 50 μm). **(D)** Immunoblotting was used to detect UCP1 protein in lysates from the iWAT, gWAT, or BAT of prolonged cold-exposed WT and TAP mice (representative image of n = 6 mice per genotype). **(E)***Fgf2*1 mRNA levels were measured from iWAT harvested from WT and TAP mice exposed for 3 days to (n = 6 per group, ∗: p < 0.05). Data are represented as mean ± SEM.

To better understand how 14-3-3ζ can improve tolerance to prolonged cold exposure, we first examined whether increased signs of beiging or BAT activity occurred as a result of 14-3-3ζ over-expression. In BAT, *Ucp1* mRNA was the same between groups, but differences in the expression of various thermogenic genes, for example, *Prdm16 and Pdk4,* were detected between WT and TAP mice (Figure 4A). In the iWAT of prolonged cold-exposed animals, a fivefold increase in *Ucp1* mRNA levels was detected in TAP mice, in addition to significantly higher levels of *Tbx1* mRNA (Figure 4B). Marked elevations in UCP1 protein abundance were also detected in the iWAT and BAT of TAP mice when compared to littermate controls (Figure 4C,D), and increased *Fgf2*1 mRNA levels were also detected in cold-exposed iWAT from TAP mice (Figure 4E). Taken together, these findings demonstrate that 14-3-3ζ over-expression can potentiate the beiging of iWAT after prolonged cold exposure.

### 3.5 Over-expression of 14-3-3ζ does not alter sensitivity to β-adrenergic stimuli or sympathetic activity

**Figure 5:**
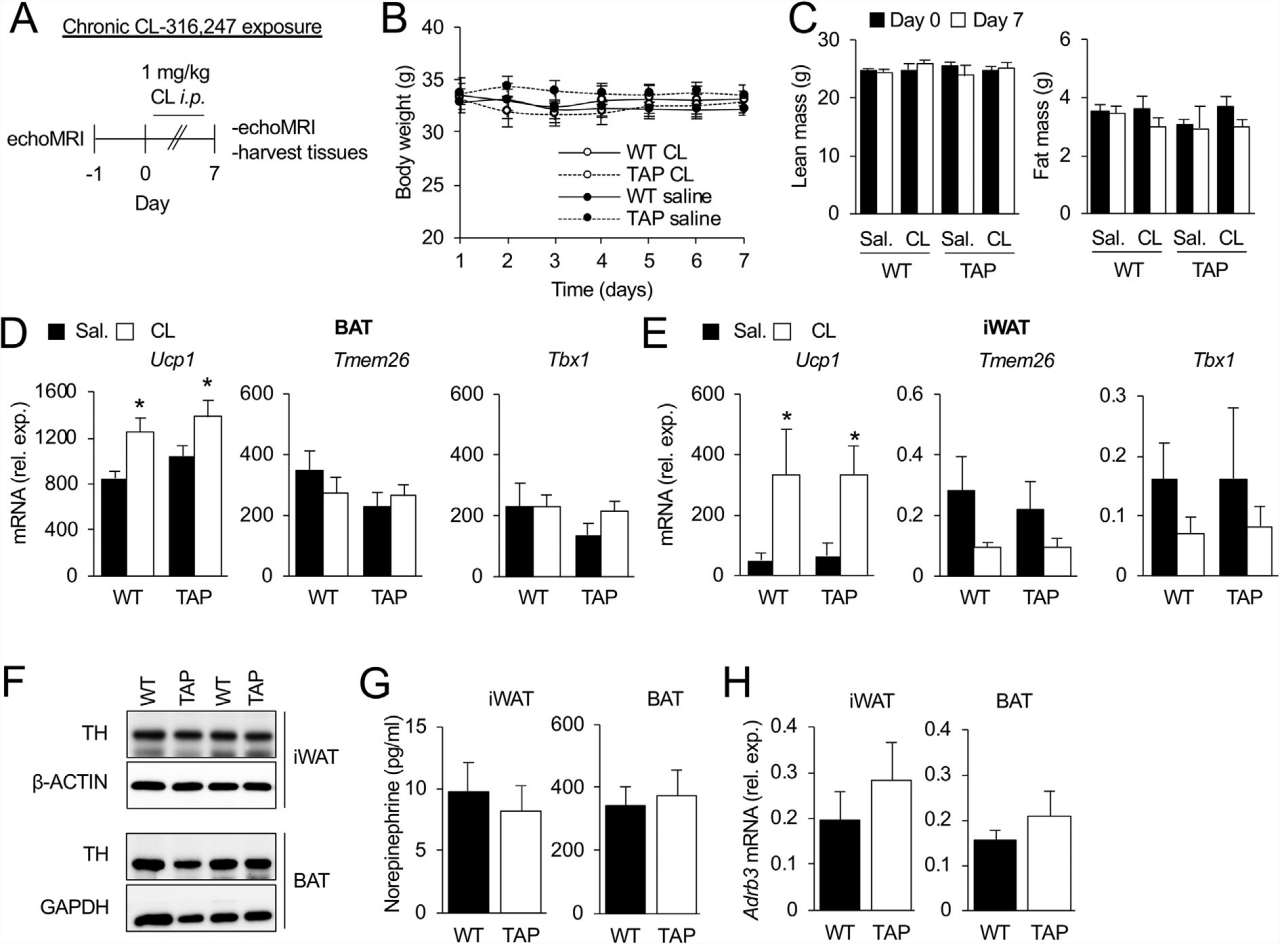
**No differences in sympathetic activity or sensitivity were detected in mice over-expressing 14-3-3ζ. (A)** To examine if 14-3-3ζ over-expression confers increased sensitivity to β-adrenergic stimuli, wildtype (WT) and transgenic 14-3-3ζ over-expressing mice (TAP) were injected with the β3-adrenergic agonist CL,316,247 (CL, 1 mg/kg) for 7 days. Lean and fat mass were measured by echo MRI before and after CL injections. **(B,C)** Bodyweight (B) and lean and fat mass (C) were obtained before and after CL treatment. **(D, E)** BAT (D) and inguinal white adipose tissue (iWAT, E) were harvested from WT and TAP mice after 7 days of injections with saline (sal.) or CL exposure to measure genes associated with thermogenesis. **(F–H)** Cold-exposed iWAT and BAT were harvested from WT and TAP mice to measure protein abundance of TH by immunoblotting (F), norepinephrine by ELISA, and *Adrb3* mRNA levels by quantiative PCR (H). (n = 5 WT and 7 TAP, ∗: p < 0.05 when compared Day = 0). Data are represented as mean ± SEM.

During long-term cold exposure, the SNS releases norepinephrine, which acts as the principal activator of thermogenesis [8,28,29,[48], [49], [50]]. Thus, to explore the possibility that altered sensitivity to β-adrenergic stimuli could account for differences in energy expenditure (Figure 2I,J), WT and TAP mice were chronically injected (7 days) with 0.9% saline or the β3-adrenergic receptor agonist CL-316,243 (CL, 1 mg/kg; Figure 5A). No differences in CL-mediated changes in total body weight, lean mass, or fat mass were observed between TAP and WT mice (Figure 5B,C). Levels of *Ucp1* mRNA were similarly increased by CL treatment in BAT (Figure 5D) and iWAT (Figure 5E) of both groups. Furthermore, markers of brown, *Cidea* and *Pdk4*, and beige, *Tmem26* and *Tbx1*, adipocytes were the same for the TAP mice and WT littermate controls (Figure 5D,E). Taken together, these data suggest that over-expression of 14-3-3ζ does not alter sensitivity to β-adrenergic stimuli.

The aforementioned *in vivo* studies demonstrate that the influence of 14-3-3ζ on cold tolerance is not due to increased sensitivity to adrenergic stimuli. Thus, we investigated whether changes in sympathetic innervation or activity in the iWAT or BAT of TAP mice could be occurring [51]. TH protein expression was not altered in the iWAT or BAT (Figure 5F) of both groups after prolonged cold exposure, and consistent with these observations, norepinephrine levels in iWAT and BAT were the similar in WT and TAP mice (Figure 5G), nor *were* mRNA levels of *Adrb3* expression in iWAT and BAT (Figure 5H). Together, these findings suggest that sympathetic innervation or activity is not altered in TAP mice in response to prolonged cold exposure.

### 3.6 14-3-3ζ does not influence glucose utilization or oxidative metabolism during prolonged cold exposure

**Figure 6:**
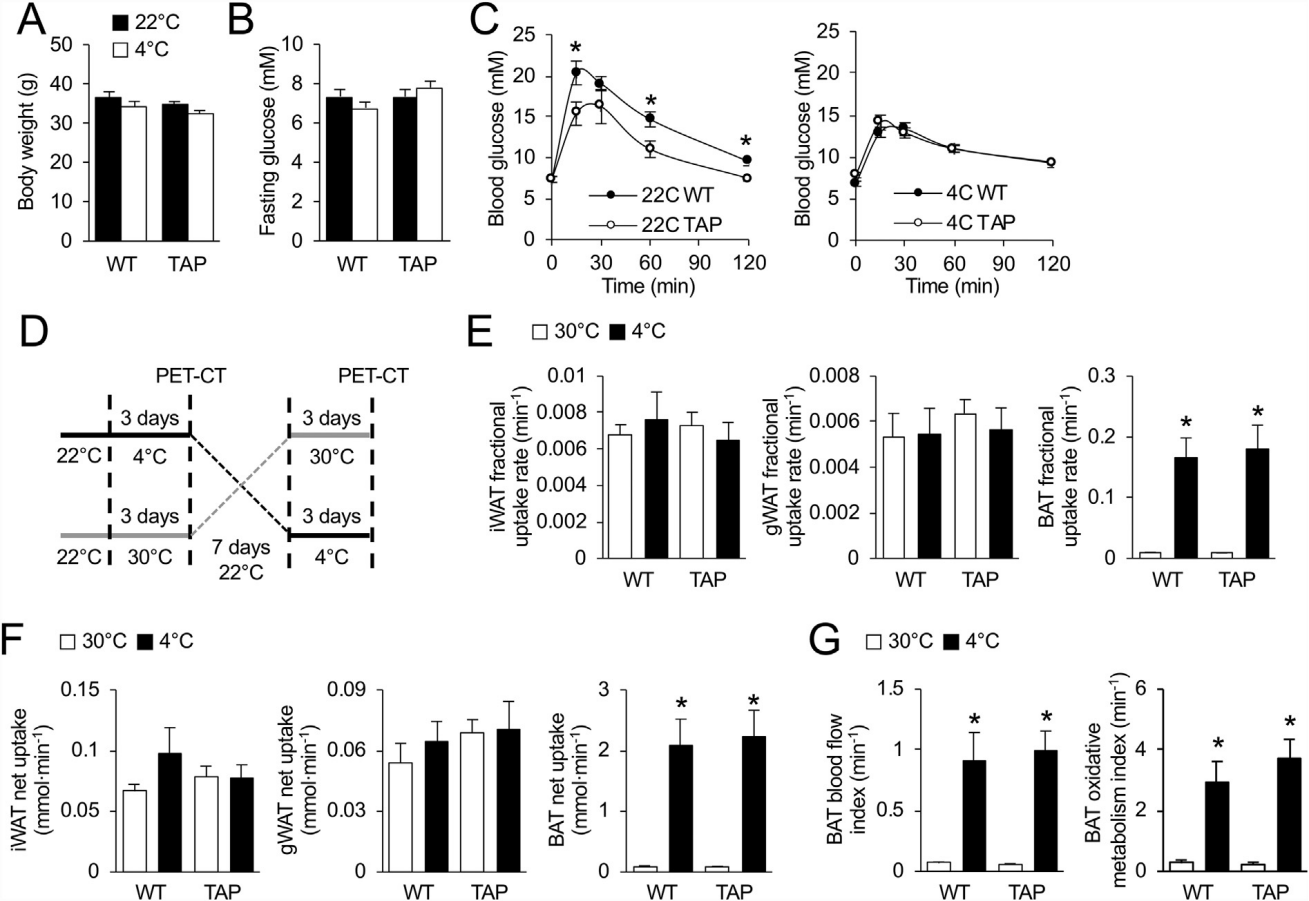
**Over-expression of 14-3-3ζ does not enhance the metabolic activity of BAT during cold exposure. (A**–**C)** Wildtype (WT) or transgenic mice over-expressing 14-3-3ζ (TAP) housed at 22 °C or 4 °C did not display differences in body weight (A) or fasting blood glucose (B) before an intraperitoneal glucose tolerance test (C, 2 g/kg) (n = 8 WT and 6 TAP; ∗: p < 0.05). **(D)** To measure the metabolic activity of iWAT and BAT during prolonged cold exposure, WT and TAP mice were subjected to a randomized crossover design whereby mice were housed at thermoneutrality (30 °C) or 4 °C for 3 days, with 1-week recovery between temperatures, and subjected to dynamic [^18^F]-FDG and [^11^C]-acetate-based PET-CT imaging. **(E,F)** Fractional and net [^18^F]-FDG uptake were measured in BAT, gWAT, and iWAT from WT and TAP mice at the indicated temperatures (n = 8 WT, 10 TAP; ∗: two-way ANOVA p < 0.05 for difference between 30 °C vs. 4 °C; ∗: two-way ANOVA p < 0.05 for difference between 30). **(G)** Oxidative activity, as measured by [^11^C]-acetate metabolism, and blood flow were measured in BAT of WT and TAP mice at the indicated temperatures (n = 8 WT, 10 TAP; ∗: two-way ANOVA p < 0.05 for difference between 30 °C vs. 4 °C). Data are represented as mean ± SEM.

Brown and beige adipocytes use triglycerides and glucose as sources of energy for heat production [9,10], and because of the differences in cold tolerance between WT and TAP mice, we explored whether glucose handling was altered by systemic 14-3-3ζ over-expression. As the first step, we examined whether differences in glucose tolerance could be detected in WT and TAP mice following a 3-day exposure of mice to 4 °C. Following a 6-hour fast, no differences in fasting body weights or blood glucose levels were observed between WT and TAP mice (Figure 6A,B). At 22 °C, TAP mice had improved glucose tolerance, but this improvement was lost after prolonged cold exposure (Figure 6C).

To better understand whether 14-3-3ζ over-expression altered glucose utilization or metabolic activity in BAT or iWAT, [^18^F]-FDG and [^11^C]-acetate-based PET-CT imaging were used. Mice were subjected to a randomized crossover study where they were housed at thermoneutrality (30 °C) or 4 °C for 3 days with one week of recovery between temperatures, followed by dynamic PET-CT imaging (Figure 6D). Aside from muscle, analysis of fractional and net [^18^F]-FDG uptake in other non-adipose tissues of mice housed at 30 °C or 4 °C revealed no differences between groups (Figure S5). When considering the glucose excursion profiles on WT and TAP mice after an *i.p.* glucose bolus (Figure 6C), these findings demonstrated that TAP mice do not depend on enhanced glucose uptake as an energy source in response to prolonged cold. Furthermore, no differences in fractional and net [^18^F]-FDG uptake were observed in BAT, iWAT, or gWAT (Figure 6E,F).

With respect to oxidative metabolism rates among non-adipose tissues, only myocardial tissue displayed decreased metabolic activity when mice were housed at 4 °C (Figure S6). Oxidative metabolism increased in the BAT of WT and TAP mice after cold exposure; however, no significant differences were observed between groups (Figure 6G). Oxidative metabolic rates in iWAT were too low for detection (data not shown). Taken together, the absence of differences in glucose uptake or metabolic activity of BAT suggest an alternative mechanism must be contributing to the ability of TAP mice to maintain body temperature during prolonged cold exposure.

### 3.7 14-3-3ζ increases vasoconstriction to mitigate heat loss

**Figure 7:**
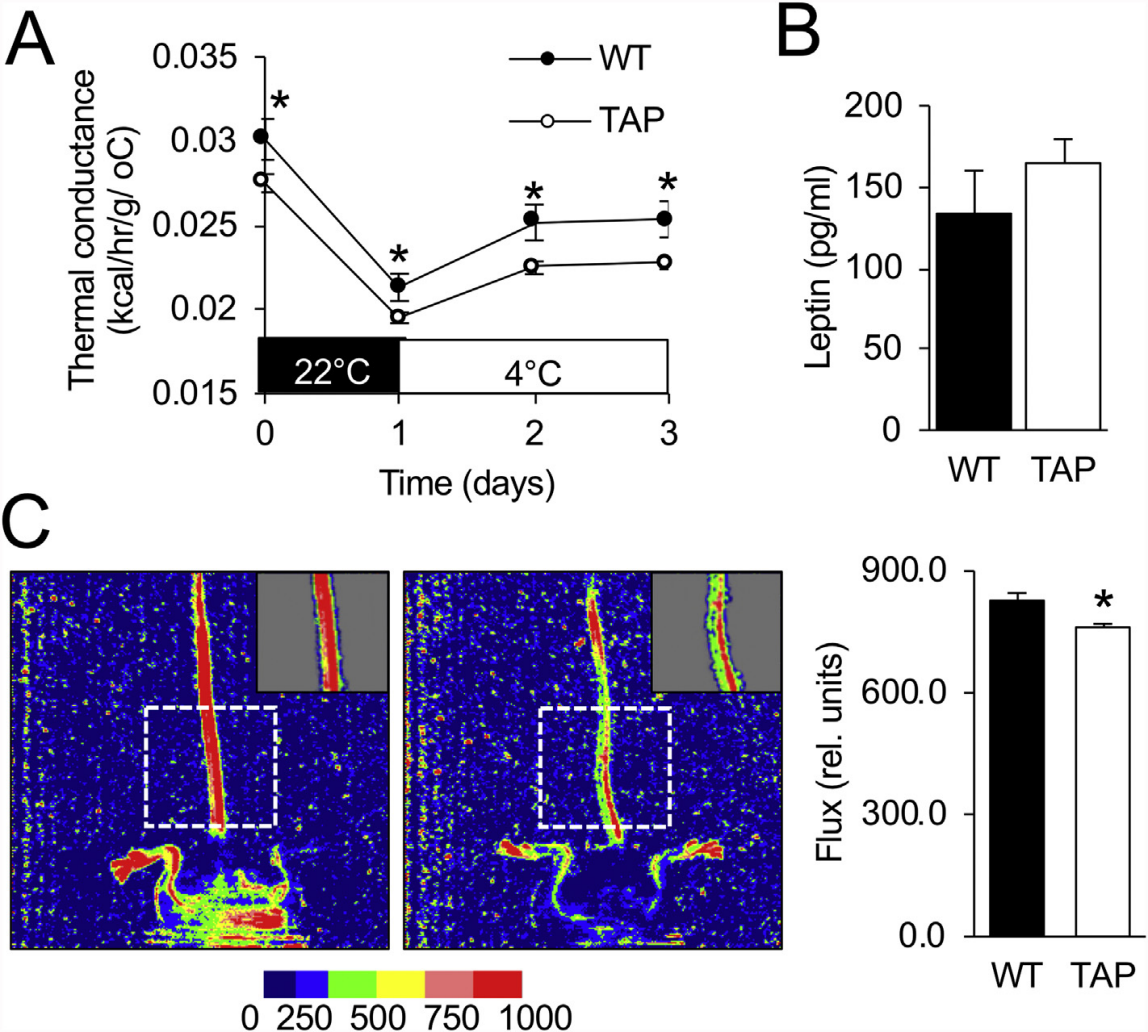
**Increased heat retention is associated with 14-3-3ζ over-expression. (A)** Thermal conductance was calculated for WT and TAP mice housed at 22 °C or 4 °C during the prolonged cold exposure CLAMS study (Figure 2; n = 8 WT and 10 TAP mice; ∗: p < 0.05 when compared to WT). **(B)** Plasma leptin was measured from blood samples obtained from WT and TAP mice after the prolonged cold challenge (n = 8 WT and 10 TAP mice). **(C)** Laser Doppler imaging was used to measure blood flow in the base of the tail of WT and TAP mice housed at 22 °C. (n = 16 WT and 22 TAP mice; ∗: p < 0.05). Data are represented as mean ± SEM.

The observed differences in cold-associated decreases in body temperature and the paradoxical decreases in energy expenditure suggest that an alternative, adaptive mechanism of heat conservation must be present in TAP transgenic mice after prolonged cold exposure. To further explore this phenomenon, thermal conductance, a measurement of the rate of heat dissipation to the environment [52], was determined in WT and TAP mice housed at 22 °C or during the prolonged cold challenge (Figure 2A). In contrast to WT mice, TAP mice displayed significantly lower thermal conductance at room temperature and throughout the prolonged cold exposure period (Figure 7A), which suggests that an alternative, separate mechanism is active in TAP mice to mitigate heat loss during mild (22 °C) and severe cold (4 °C) stress. Leptin has been found to have thermo-regulatory effects in *ob/ob* mice; however, circulating levels of leptin were the same in WT and TAP mice (Figure 7B) [35,52,53]. The tail of rodents represents a major site of heat loss because up to 25% of heat can be dissipated from this site, and constriction and dilation of vessels in the tail regulates heat loss [54]. Thus, to explore whether 14-3-3ζ over-expression could affect vasoconstriction *in vivo,* laser Doppler imaging was used to measure superficial blood flow as a surrogate measure of vessel diameter [37]. At 22 °C, blood flow was significantly reduced in the TAP animals (Figure 7C), which suggests increased vasoconstriction. Taken together, these findings suggest that over-expression of 14-3-3ζ in mice prevents heat loss, potentially via increased vasoconstriction in the tail.

## 4 Discussion

Substantial interest in understanding the roles of beige and brown adipocytes in the regulation of homeothermy and energy homeostasis has occurred over the past decade, and due to their abilities to metabolize lipids via β-oxidation, this has sparked interest in exploring the possibility of activating beige and brown adipocytes as a therapeutic approach to treat obesity and diabetes. In the context of homeothermy, a general assumption is that UCP-1-dependent thermogenic pathways are necessary to protect against cold, and little emphasis has been placed on alternative, UCP-1-independent mechanisms that may also influence cold tolerance. Herein, we demonstrate that over-expression of 14-3-3ζ is sufficient to improve tolerance to acute and prolonged cold exposure. Transgenic mice over-expressing 14-3-3ζ better defended decreases in their body temperature following cold exposure, and this physiological response was associated with a parallel decrease in energy expenditure and the restriction of peripheral blood flow to retain heat. By minimizing heat loss, less energy has to be expended to defend against hypothermia and maintain body temperature. Taken together, these findings demonstrate that despite the activation of thermogenic mechanisms after cold exposure, alternative mechanisms that promote heat conservation must also be considered in the context of cold tolerance.

Adaptive mechanisms to maintain core body temperature are activated to defend against cold exposure, and this predominantly results in the production of heat via UCP1-dependent mechanisms in brown and beige adipocytes [1,8]. However, in the present study, the improved tolerance of transgenic mice over-expressing 14-3-3ζ in response to prolonged cold was associated with decreased energy expenditure, with transgenic mice being able to maintain higher body temperatures. Notably, intact adaptive thermogenic mechanisms are present in TAP mice because the expected increases in UCP1 expression in BAT after cold exposure could be detected, as well as the elevated oxidative metabolism of BAT. Thus, an alternative mechanism to defend against changes in core body temperature that are independent of BAT activity must be active in transgenic mice over-expressing 14-3-3ζ.

Thermogenesis is largely considered to be the primary mechanism for cold tolerance, but when this process is disrupted, as in the case of the UCP1-deficient mice, vasoconstriction becomes the predominant mechanism for cold adaptation [7]. The decrease in thermal conductance during mild (22 °C) and severe (4 °C) cold stress raised the possibility that 14-3-3ζ over-expression could activate processes, for example, vasoconstriction, to mitigate heat loss from skin [52]. Acute cold exposure was found to increase mRNA levels of *Ywhaz* in iWAT and BAT, which suggests that a similar phenomenon could occur in endothelial cells, but whether physiological increases in 14-3-3ζ protein directly translate to effects on vasoconstriction requires further examination. Nonetheless, we found that transgenic over-expression of 14-3-3ζ was associated with reduced peripheral blood flow in the tails of TAP mice, which is indicative of vasoconstriction [7]. Leptin has been recently identified as having vasoconstrictive effects, but in this study, no differences in circulating leptin levels were detected between WT and TAP mice [35,52,53]. A possibility is that the increase in vasoconstriction could be mediated in part by 14-3-3ζ influencing the actions of the potent vasoconstrictor norepinephrine at endothelial cells. For example, 14-3-3ζ has been found to interact with α2A-adrenergic receptors subtypes at the i3 loop, which blocks their interactions with β-arrestins to limit their internalization. Increased receptor expression at the cell membrane of endothelial cells could result in potentiated α2A-adrenergic receptor-mediated effects on vasoconstriction [55,56]. It is unclear why 14-3-3ζ over-expression results in decreased energy expenditure only during the dark cycles of the prolonged cold challenge (Figure 2I,J and Figure S4), but mean arterial pressure, which is partly regulated by vasoconstriction, and circulating catecholamines are known to follow diurnal rhythms, whereby both are higher in the dark, active phase of mice [[57], [58], [59], [60]]. Thus, 14-3-3ζ over-expression may potentiate norepinephrine-mediated effects on arterial pressures by promoting vasoconstriction specifically during the dark phase to reduce heat loss.

In summary, we report in this study that over-expression of 14-3-3ζ in male mice is sufficient to increase tolerance to cold. Despite the activation of thermogenic mechanisms, increasing levels of 14-3-3ζ reduce the decrease in core body temperature after cold exposure, and this was paradoxically associated with decreased energy expenditure in mice over-expressing 14-3-3ζ and increased peripheral vasoconstriction to retain heat. These findings suggest that alternative, non-thermogenic mechanisms can also be activated to defend against cold and raise the notion that other mechanisms, which are equally important in the defence of homeothermy and should be considered when studying cold tolerance. Taken together, our results show that 14-3-3ζ is capable of exerting a strong influence on cold tolerance and provide new insights into how molecular scaffolds participate in physiological processes.

## Contributions

K.D. designed the studies, conducted the research, interpreted the results, and wrote the manuscript.

A.F.L. provided the 14-3-3ζ HET mice. S.D. and A.R designed and conducted laser Doppler analysis of blood flow and revised the manuscript. C.N and A.C.C. designed the experiments, analyzed the data, and wrote the manuscript. G.E.L conceived of the concept, designed the studies, interpreted the results, and wrote and revised the manuscript. G.E.L is the guarantor of this work.
