# Supplementary material for: 14-3-3ζ mediates an alternative, non-thermogenic mechanism in male mice to reduce heat loss and improve cold tolerance
Source: Mol Metab. 2020 Jul 12;41:101052. doi: 10.1016/j.molmet.2020.101052 (PMC7394917; doi:10.1016/j.molmet.2020.101052)
Supplement: Multimedia component 1 [file mmc1.pdf]

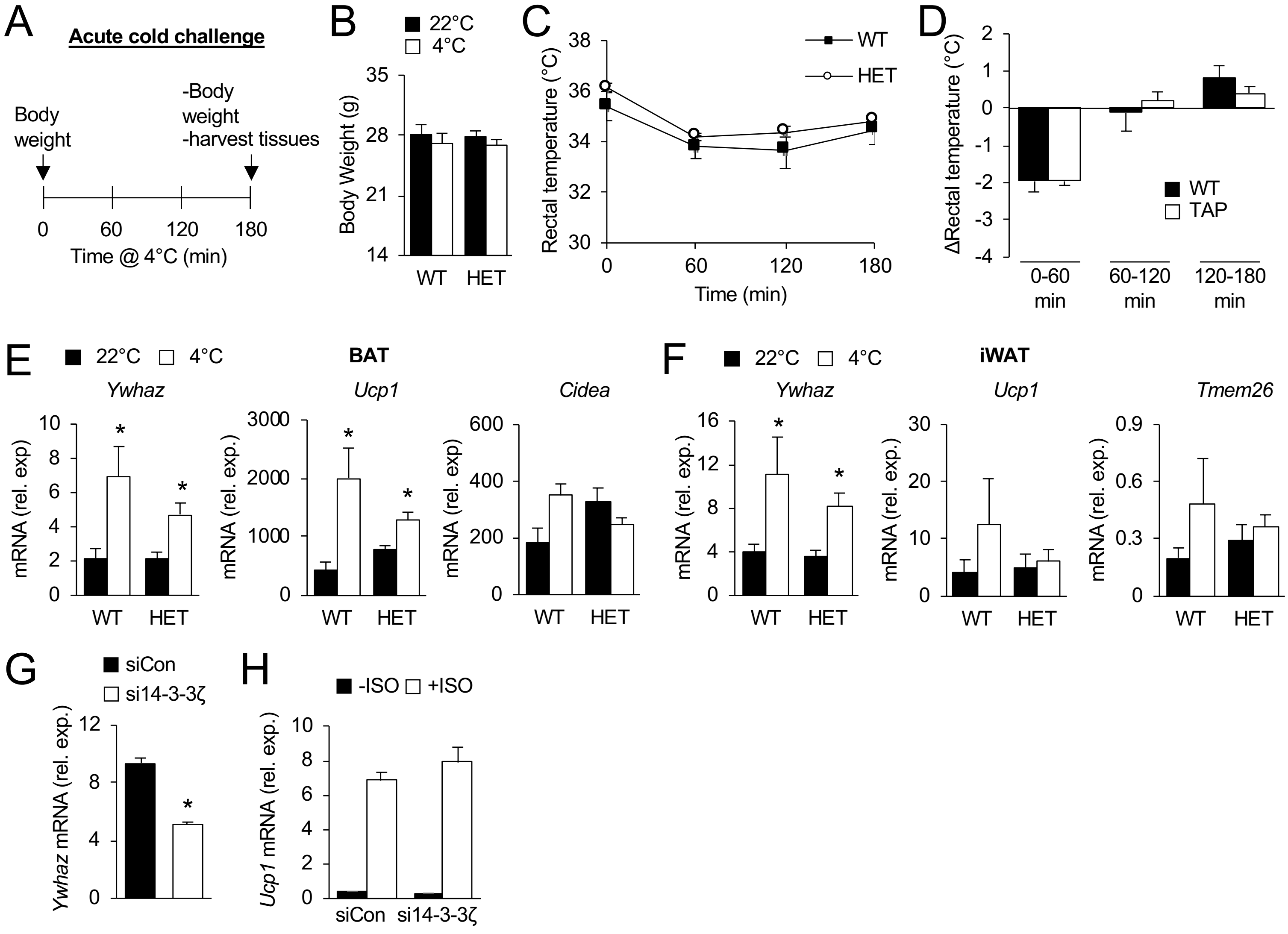

Figure S1- Loss of one allele of *Ywhaz* does not affect tolerance to acute cold in male mice. (A) Wildtype (WT) and mice lacking one allele of *Ywhaz*, the gene encoding 14-3-3ζ (HET) were challenged with cold for 3 hours. Temperature was measured by rectal probes at each time point. **(B-D) (A-C)** Body weights (B), rectal temperatures (C), and change (Δ) in rectal temperature (D) of WT and HET mice were obtained prior, during, and at the end of the 3 hours cold challenge (n=7 mice per group). **(E,F)** Expression of brown-selective (D) and beige-selective (E) genes from BAT and iWAT, respectively, at room temperature (22 °C) and after 3 hours cold exposure (n=7 per group, *: $p<0.05$ when compared to 22 °C). **(G,H)** Knock-down of 14-3-3ζ expression by siRNA (G) in UCP1-luciferase (UCP1-Luc) cells, a brown adipocyte cell line, does not affected isoproterenol (ISO, 10μM, 4 hours)-mediated induction of *Ucp1* expression (H) (n=6 per group, *: $p<0.05$ when compared to -ISO; #: $p<0.05$ when compared to siCon). Data are represented as mean ± SEM.

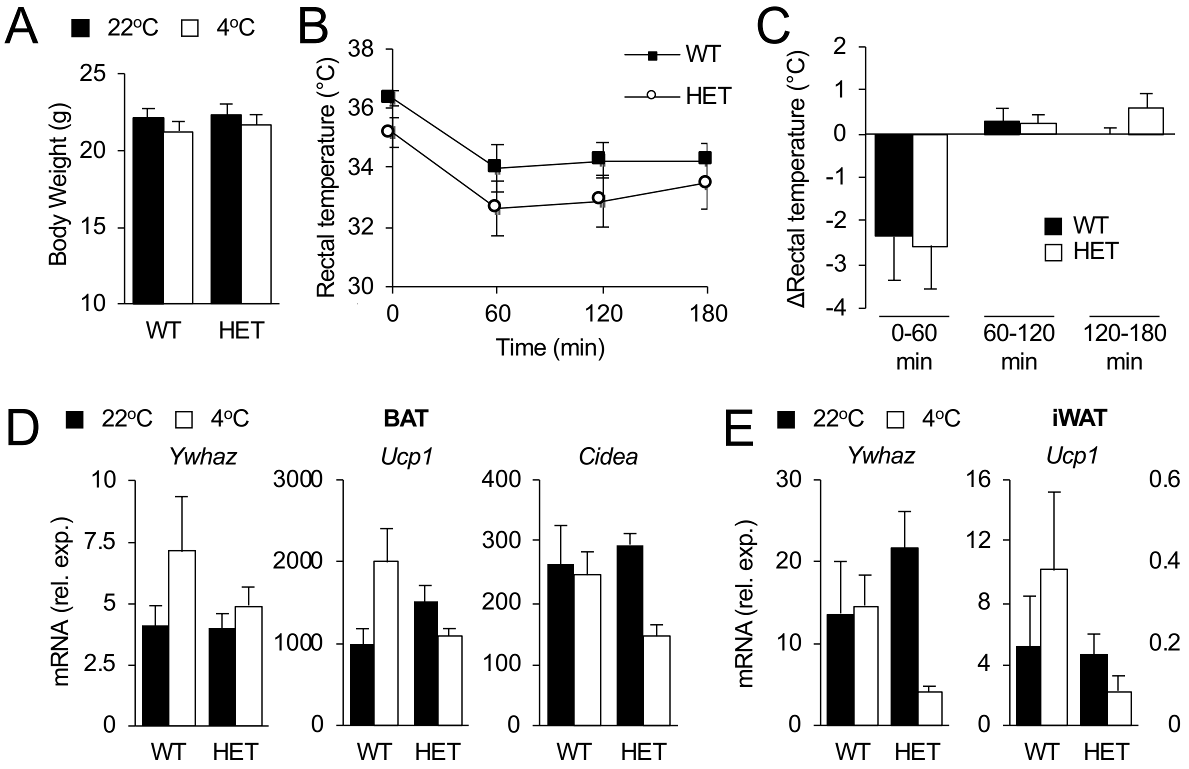

Figure S2- Reducing 14-3-3ζ expression in female mice does not impact acute cold tolerance (A-C) Body weights (A), rectal temperatures, and change (Δ) in rectal temperature (C) of female WT and HET mice were obtained prior, during, and at the end of the 3 hour cold challenge (n=7 mice per group). **(C,D)** Expression of brown-selective (C) and beige-selective (D) genes from BAT and iWAT, respectively, at room temperature (22 °C) and after 3 hours cold exposure (n=7 per group, *: p<0.05 when compared to 22 °C).

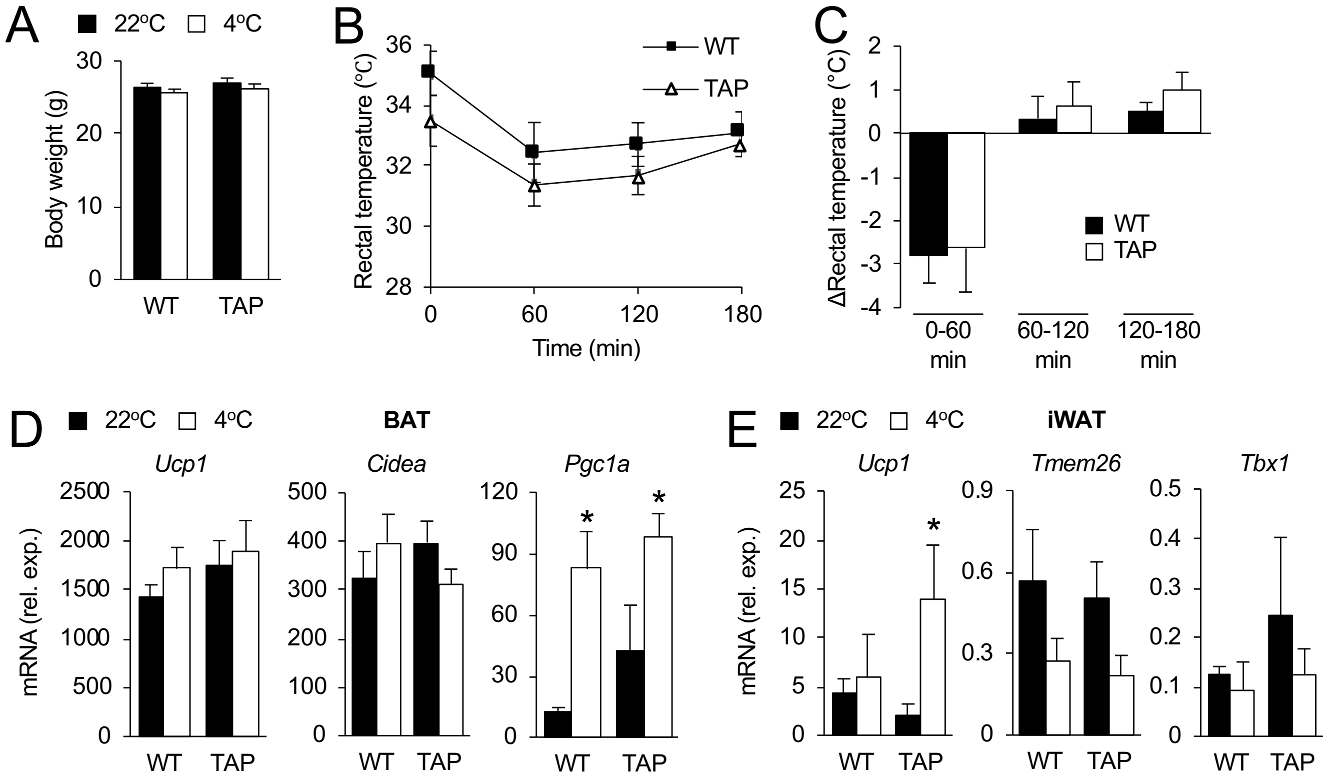

Figure S3- Over-expression of 14-3-3ζ in female mice does not impact acute cold tolerance. (A-C) Body weights (A), rectal temperatures, and change (Δ) in rectal temperature (C) of WT and TAP mice were obtained prior, during, and at the end of the 3 hour cold challenge (n=7 mice per group). **(D,E)** Expression of brown-selective (D) and beige-selective (E) genes from BAT and iWAT, respectively, at room temperature (22 °C) and after 3 hours cold exposure (n=7 per group, *: $p<0.05$ when compared to 22 °C).

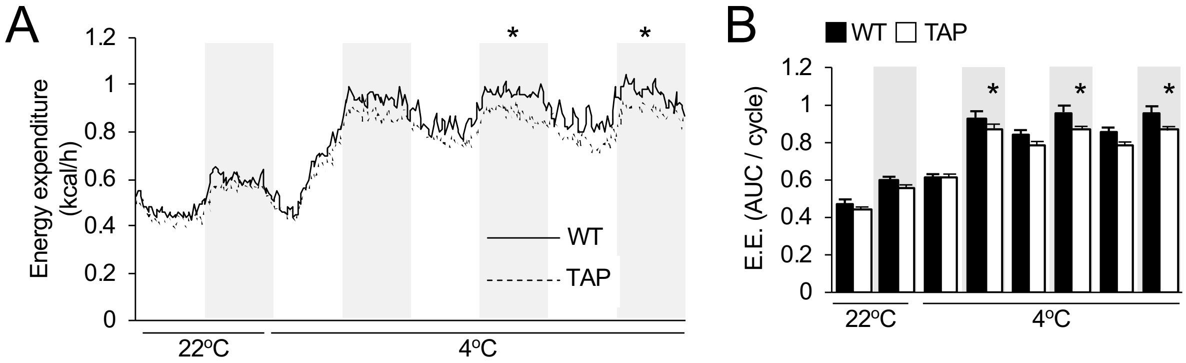

Figure S4. Whole-body energy expenditure during the prolonged cold challenge. (A) Visualization of whole-body energy expenditure of WT and TAP mice from Figure 3I during the prolonged cold challenge. Data are reported either as the average trace for all mice over the cold challenge (A) or as the area under the curve (AUC) per group per light:dark cycle (B) (n=8 WT and n=10 TAP mice; *: $p<0.05$ when compared to WT). Data are represented as mean ± SEM

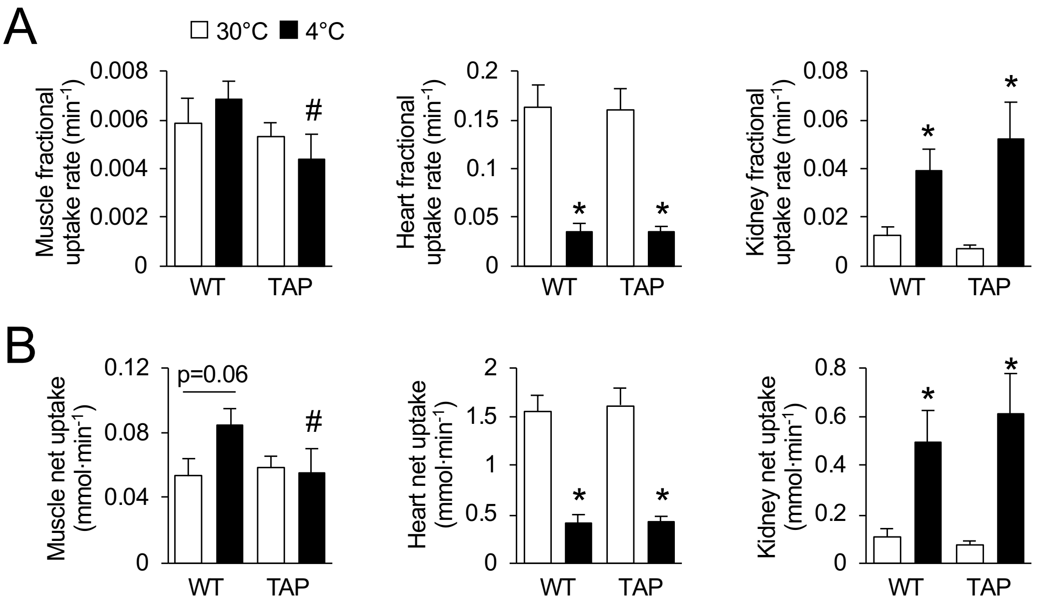

Figure S5: **14-3-3ζ over-expression does not alter [^{18}F]-FDG uptake in the heart or kidney of cold exposed mice.** Following exposure to thermoneutrality (30°C) or 4°C for 3 days, Fractional (A) and net (B) [^{18}F]-FDG uptake was measured in muscle, heart, and kidneys from WT and TAP mice at the indicated temperatures (n=8 WT, 10 TAP; *: $p<0.05$).

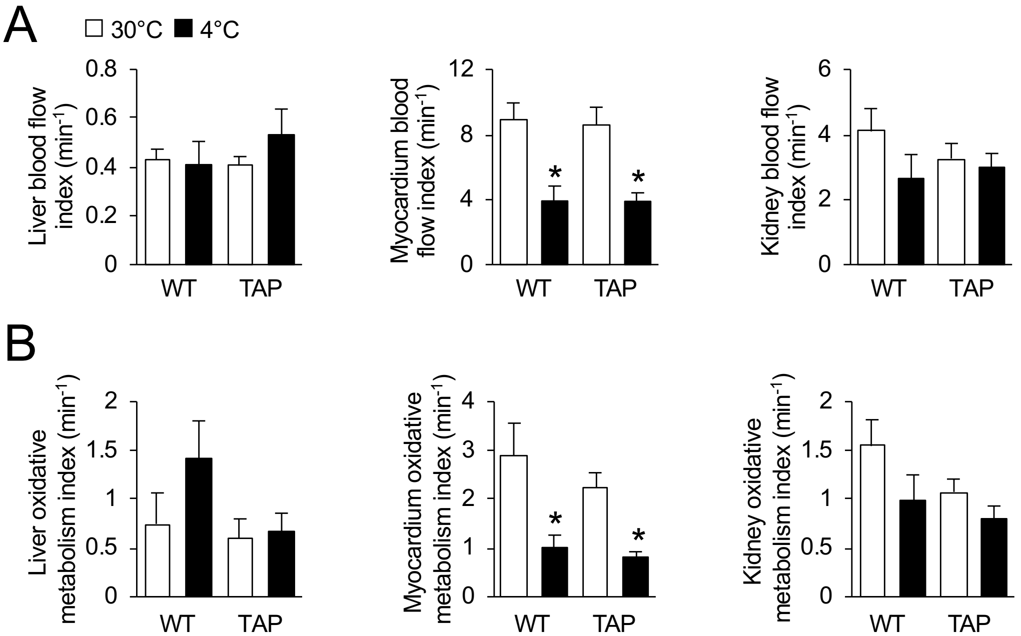

Figure S6: No differences in oxidative metabolism or blood flow were detected in non-adipose tissues following cold exposure. After being housed at thermoneutrality (30°C) or 4°C for 3 days, blood flow (A) and oxidative activity, as measured by [^{11}C]-acetate metabolism (B), were measured in myocardium, liver, and kidneys of WT and TAP mice at the indicated temperatures (n=8 WT, 10 TAP; *: $p<0.05$).

Table S1: List of Antibodies

	Compay	Dilution	Product number	RRID
Alexa Fluor 594	Jackson Immunoresearch	1:400	115-585-003	AB_2338871
Anti-Mouse HRP IgG	Cell signaling	1:5000	7076S	AB_330924
β-Actin	Cell signaling	1:10000	3700S	AB_2242334
β-Tubulin	Cell signaling	1:10000	86298S	AB_2715541
GAPDH	Cell signaling	1:10000	5174S	AB_10622025
Perilipin	Cell signaling	1:400	9349S	AB_10829911
TH	Millipore	1:400	MAB318	AB_2201528
TH	Millipore	1:400	AB152	AB_390204
UCP1	ABCAM	1:1000	Ab10983	AB_2241462
UCP1	R&D systems	1:400	MAB6158-SP	AB_10572490

Table S2: List of primers for qPCR

Gene	Forward	Reverse	Ref.
Adrb3	CCTTCAACCCGGTCATCTAC	GAAGATGGGGATCAAGCAAGC	1
Cidea	TGCTCTTCTGTATCGCCCAGT	GCCGTGTTAAGGAATCTGCTG	2
Fgf21	CTGGGGGTCTACCAAGCATA	CACCCAGGATTTGAATGACC	3
Hprt1	TCCTCCTCAGACCGCTTTT	CCTGGTTCATCATCGCTAATC	4
Pdk4	CCGCTTAGTGAACACTCCTTC	TCTACAAACTCTGACAGGGCTTT	2
Pgc1a	AGCCGTGACCACTGACAACGAG	GCTGCATGGTTCTGAGTGCTAAG	2
Pparg2	GTTATGGGTGAAACTCTGGGAGAT	GGCCAGAATGGCATCTCTGTGTCAA	4
Prdm16	CAGCACGGTGAAGCCATTC	GCGTGCATCCGCTTGTG	2
Tbx1	GGCAGGCAGACGAATGTTC	TTGTCATCTACGGGCACAAAG	2
Tmem26	ACCCTGTCATCCCACAGAG	TGTTTGGTGGAGTCCTAAGGTC	2
Ucp1	ACTGCCACACCTCCAGTCATT	CTTTGCCTCACTCAGGATTGG	2
Ywhaz	CAGAAGACGGAAGGTGCTGAGA	CTTTCTGGTTGCGAAGCATTGGG	5
YWHAZ	ACCGTTACTTGGCTGAGGTTGC	CCCAGTCTGATAGGATGTGTTGG	5

1. Lee Y, Petkova AP, Konkar AA, Granneman JG. Cellular origins of cold-induced brown adipocytes in adult mice. FASEB J, 29(1) (2015) 286-299.

2. Wu J, Boström P, Sparks LM, Ye L, Choi JH, Giang AH, Khandekar M, Virtanen KA, Nuutila P, Schaart G, Huang K, Tu H, van Marken Lichtenbelt WD, Hoeks J, Enerbäck S, Schrauwen P, Spiegelman BM. Beige adipocytes are a distinct type of thermogenic fat cell in mouse and human. Cell, 150(2) (2012) 366-76.

3. Jornayvaz FR, Birkenfeld AL, Jurczak MJ, Kanda S, Guigni BA, Jiang DC, Zhang D, Lee HY, Samuel VT, Shulman GI. Hepatic insulin resistance in mice with hepatic over-expression of diacylglycerol acyltransferase 2. Proc Natl Acad Sci U S A., 108(14) (2011) 5748-52.

4. Mugabo Y, Sadeghi M, Fang NN, Mayor T, Lim GE. Elucidation of the 14-3-3ζ interactome reveals critical roles of RNA-splicing factors in adipogenesis. J Biol Chem, 293(18) 6736-50.

5. Lim GE, Piske M, Johnson JD. 14-3-3 proteins are essential signalling hubs for beta cell survival. Diabetologia, 56(4) (2013) 825-37.
